# Predominance of Non-carbapenemase Producing Carbapenem-Resistant Enterobacterales in South Texas

**DOI:** 10.3389/fmicb.2020.623574

**Published:** 2021-02-10

**Authors:** Cody A. Black, Wonhee So, Steven S. Dallas, Gerard Gawrys, Raymond Benavides, Samantha Aguilar, Chang-Jui Chen, James F. Shurko, Grace C. Lee

**Affiliations:** ^1^The University of Texas at Austin, College of Pharmacy, Austin, TX, United States; ^2^School of Medicine, The University of Texas Health San Antonio, San Antonio, TX, United States; ^3^Department of Pathology and Laboratory Medicine, The University of Texas Health San Antonio, University Health System, San Antonio, TX, United States; ^4^School of Pharmacy, Long Island University, Brooklyn, US and Pharmacy, New York Presbyterian/Weill Cornell Medical Center, New York, NY, United States; ^5^Methodist Healthcare System, San Antonio, TX, United States

**Keywords:** carbapenem, resistance, epidemiology, outcomes, Enterobacterales

## Abstract

**Background:**

Carbapenem-resistant Enterobacterales (CRE) pose a significant global public health threat. Resistance among CRE is particularly complex, owing to numerous possible resistance mechanisms and broad definitions. We aimed to characterize the clinical and molecular profiles of CRE in the South Texas region.

**Materials and methods:**

We compared the clinical, genotypic, and phenotypic profiles of carbapenemase producing Enterobacterales (CPE) with those of non-carbapenemase producers (NCPE) isolated from South Texas, United States between 2011 and 2019. Molecular characteristics and resistance mechanisms were analyzed using whole-genome sequences.

**Results:**

The majority (59%) of the CRE isolates were NCPE while 41% of isolates harbored carbapenemases, predmonantly *bla*_KPC_-type. The most common CPE was *Klebsiella pneumoniae* while majority of *Enterobacter cloacae* and *Escherichia coli* were NCPE Among *K. pneumoniae*, the clonal group 307 has emerged as a predmoninant group and was associated with as many CRE infections as the previous common clonal group 258. Patients with NCPE compared to CPE infections were associated with higher antimicrobial exposure prior to culture collection (days of therapy, 795 vs. 242; *p* < 0.001) and emergency department visits within past 90 days (22% vs. 4%; *p* = 0.011). The all cause 30-day mortality was 21%.

**Conclusions:**

This study highlights the diversity of resistance mechanisms underlying CRE in South Texas, with 59% not harboring a carbapenemase. Individuals with NCPE infections were more likely to have had prior antimicrobial therapy and emergency department visits compared to those with CPE. Identification and distinction of these mechanisms by rapid identification of species and carbapenemase would allow for optimal treatment and infection control efforts.

## Introduction

Carbapenem-resistant Enterobacterales(CRE), formerly known as Enterobacteriaceae, are a group of organisms that are among the most difficult to treat. It was given a level one priority by the World Health Organization in 2017 (WHO, 2020), and also remains one of the top five most urgent public health threat in 2019 by U.S. Centers of Disease Control and Prevention (Centers for Disease Control and Prevention (U.S.), 2019).

The broad definition of “CRE” remains ambiguous. For example, CREs can include those due to porin-deficient Enterobacterales, Enterobacterales with intrinsic imipenem resistance, and Enterobacterales with carbapenemase enzymes that remain “susceptible” to carbapenems. Globally, common carbapenemases in Enterobacterales include the *Klebsiella pneumoniae* carbapenemases (KPC), oxacillinase (OXA)-48-like β-lactamases, and metallo-β-lactamases, such as New-Delhi-metallo-β-lactamases (NDM), the active-in-imipenem family of carbapenemases, and Verona integron-encoded metallo-β-lactamases (VIM). In the U.S., KPC is the most prevalent type, however; sporadic outbreaks of class B (e.g., VIM, NDM, IMP) or class D (e.g., OXA-48) β-lactamase infections have also occurred (Chea et al., 2015). Importantly, emerging reports indicate that a subset of CDC-defined CRE in the United States do not produce carbapenemases (Duin et al., 2020). Specifically, carbapenem resistance *in K. pneumoniae* can occur from the production of extended-spectrum β-lactamases (ESBLs) and/or AmpC enzymes in combination with decreased antibiotic intracellular accumulation by mutations in outer membrane proteins OmpK35 and OmpK36 or over-expression of efflux pumps.

Treatment of CRE infections remains challenging for clinicians since it encompasses a wide range of genera, different resistant mechanisms requiring dissimilar treatment options, and variable local and global epidemiology. Distinguishing between these different mechanisms, as well as identification of the carbapenemase type, warrants further investigation as these have important treatment implications, particularly for newer β-lactam/β-lactamase inhibitor combinations. Herein, we characterized clinical, genotypic, and phenotypic profiles of sequentially collected CRE isolates from South Texas, United States to investigate the epidemiologic characteristics. We compared mechanisms and implications among Enterobacterales that are carbapenemase producers (CPE) with those that are non-carbapenemase producers (NCPE).

## Results

### Microbiological Characteristics

We identified 99 CRE isolates collected from 85 patients. Most of the infections were caused by *K. pneumoniae* (43%), followed by *Escherichia coli* (27%), *Enterobacter cloacae* (12%), *Klebsiella aerogenes* (7%), *Citrobacter freundii* (3%), *Serratia marcescens* (3%), *Klebsiella michiganensis* (2%), and *Morganella morganii* (2%), and *Hafnia alvei* (1%). Carbapenemase genes were present in 42 (41%) of CRE, referred to as CPE. In 58 (59%), *in vitro* resistance to at least one carbapenem was confirmed in the absence of any carbapenemase gene, referred as NCPE. There were 17 isolates that were unconfirmed-CRE (identified initially as carbapenem-resistant by local laboratories, but were found to be susceptible or intermediate to all tested carbapenems upon repeat testing) (Table 1).

**TABLE 1:** Distribution of carbapenemase and non-carbapenemase producing Enterobacterales.

	*bla*_KPC_	*bla*_NDM_	*bla*_OXA_-type^b^	Other CPs^c^	CPE	NCPE	Unconfirmed
Number of isolates^a^ *n* = 99; *n* (%)	30 (30)	5 (5)	2 (2)	5 (5)	41 (41)	58 (59)	17 (17)
Bacterial species; *n* (%)							
*Klebsiella pneumoniae*^a^ (*n* = 41)	20 (49)	2 (5)	2 (5)	2 (5)	26 (63)	16 (39)	6 (15)
*E. cloacae complex* (*n* = 11)	1 (9)	1 (9)	0	0	2 (18)	9 (82)	1 (9)
*Escherichia coli* (*n* = 28)	5 (18)	2 (7)	0	0	7 (25)	22 (79)	7 (25)
*Klebsiella aerogenes* (*n* = 7)	2 (29)	0	0	0	2 (29)	5 (71)	1 (14)
*Citrobacter freundii* (*n* = 3)	1 (33)	0	0	0	1 (33)	2 (67)	0
*Serratia marcescens*^a^ *(n* = 3)	1 (33)	0	0	3 (100)	3 (100)	0	0
*Morganella morganii* (*n* = 2)	0	0	0	0	0	2 (100)	1 (50)
*Klebsiella michiganensis* (*n* = 2)	0	0	0	0	0	2 (100)	1 (50)
*Hafnia alvei* (*n* = 1)	0	0	0	0	0	1 (100)	0

*CP, carbapenemases; KPC, Klebsiella pneumoniae carbapenemase; NCPE, non-carbapenemase producing carbapenem-resistant Enterobacterales, NDM, New Delhi metallo-β-lactamase; OXA, oxacillinase.^*a*^A patient may carry more than one type of CPE. ^*b*^OXA-type includes OXA-48 and OXA-232. ^*c*^Other CP types include IMI-like, IMP-like, SME and VIM.*

The most common source of CRE isolates was urine [35 (36%)], followed by respiratory [15 (15%)], wound tissue/bone [11 (11%)], and blood [8 (8%)]. Individuals with CRE infections with NCPE were more likely to have respiratory infections compared with patients with CPE (*p* = 0.044). There were similar distributions of CPE and NCPE associated with urinary, blood, and wound infections (Table 2). Distribution by species are further described in Supplementary Table 1.

**TABLE 2:** Distribution of carbapenemase and non-carbapenemase producing Enterobacterales and source of infection.

	*bla*_KPC_	*bla*_NDM_	*bla*_OXA_-type	Other CPs	NCPE	*P (CPE vs. NCPE)*
Urine	14	1	1	0	12	0.0557
Wound swab	1	0	0	0	0	∼
Bone and soft tissues	4	1	0	0	6	0.773
Blood	1	0	1	0	6	0.325
Hepatobiliary specimen	1	0	0	0	6	0.123
Abdominal specimen	3	0	0	0	3	0.659
Respiratory specimens	3	0	0	0	12	0.044

*^*a*^Source of infection was not provided identified in 17 isolates.*

### Molecular and Antimicrobial Susceptibility Characteristics

Resistance mechanisms were evaluated for CRE isolates (Table 1). KPC-type enzymes were the most common carbapenemases and were identified in 30 isolates (30.3%). Observed carbapenemase genes included *bla*_KPC–2_ [21 (21%) of 99], *bla*_KPC–3_ [8 (8%)], *bla*_NDM_ [5 (5%)], and *bla*_OXA–232_-like [2 (2%); Table 1]. One *S. marcescens* isolate was found to simultaneously carry *bla*_KPC–2_ and *bla*_SME–3_. Additionally, it harbored *bla*_SRT–2_. Extended spectrum β-lactamase genes were commonly found in CPE including *bla*_CTX–M_ [17 (42%)] and *bla*_SHV_ [9 (22%) of 41]. NCPE isolates predominantly carried *bla*_CTX–M_ [28 (48%) of 59]. Among NCPE, there were no differences in the rate of *bla*_AmpC_ carriage compared to CPEs [12 (20%) vs. 5 (12%); *p* = 0.15] and unconfirmed CRE [4 (20%); *p* = 0.21]. Among *K. pneumoniae* isolates, mutations in genes encoding outer membrane porins OmpK35 and OmpK36 were present in 62% of NCPE and 53% of unconfirmed CRE. The main mutations included premature stop codons and Gly115-Asp116 insertion in the gene encoding OmpK36. The majority of *Klebsiella* contained several mutations in OmpK37. Specifically, I70M, I128M, N230G, M233Q, T234H, Q235Y, N237H, R239K, E244D, N274S, D275T, and V277I were observed, presumably slowing the permeation of β-lactams through the channel. However, these implications are less defined.

Antimicrobial susceptibility results are shown in Table 3. Among the non-β-lactam-inhibitor antimicrobial agents that serve as candidates for the treatment of CRE, tigecycline, amikacin, and colistin showed the highest rates of susceptibility of 97, 91, and 89% respectively. Comparatively, CPE maintained higher susceptibility to older generation tetracyclines (doxycycline and minocycline) compared to NCPE (69 and 89% vs 41 and 52%, respectively); whereas, NCPE isolates maintained higher susceptibility to cefepime compared to CPE (41% vs. 19%). Polymyxin resistance was detected in two *K. pneumoniae*, one *E. coli*, and one *H. alvei* isolate in addition to the intrinsic resistance displayed for *Serratia* and *Morganella* spp. Upon repeat susceptibility testing, susceptibility for ertpapenem and meropenem were 18 and 52%, respectively. All but one of the isolates that were classified as unconfirmed-CRE were NCPE.

**TABLE 3:** Antimicrobial susceptibilities.

Name	All (%)	CPE (%)	NCPE (%)
AMI	91	91	91
AZT	11	9	12
CIP	19	16	21
DOX	54	69	41
ERT	18	2	28
FEP	26	19	41
FOT	11	16	9
GEN	51	44	53
MEM	52	22	69
MIN	63	81	52
TAZ	17	16	17
TGC	97	97	97
TOB	43	41	45
TZP	27	22	29

*CPE, carbapenemase-producing Enterobacterales (*n* = 38), NCPE, non-carbapenemase-producing Enterobacterales (*n* = 58); AMI, Amikacin; AZT, Aztreonam, CIP, Ciprofloxacin; DOX, Doxyclicline; ERT, Ertapenem; FEP, Cefepime; FOT, Cefotetan; GEN, Gentamicin; MEM, Meropenem; MIN, Minocycline; TAZ, Ceftazidime; TGC, Tigecycline; TOB, Tobramycin; TZP, Piperacillin-tazobactam.*

The distribution of resistance determinants among CPE, NCPE and unconfirmed strains are summarized in Supplementary Tables 2, 3. *K. pneumoniae* strains carried more *bla*_KPC_, *bla*_CTX–M_-variants, *bla*_SHV_-variants, *fosA*-like, and *oqxA/B* genes compared with other Enterobacterales including *E. coli*. On the other hand, more *bla*_pAmpC_ were prevalent in other Enterobacterales than those in *K. pneumoniae* strains. Notably, IncHI2-ST1 plasmids harboring the *mcr-9* gene was identified in 3 isolates (*E. cloacae* and *K. michiganensis*). Interestingly, these 3 isolates did not display *in-vitro* polymyxin non-susceptibility.

**FIGURE 1:**
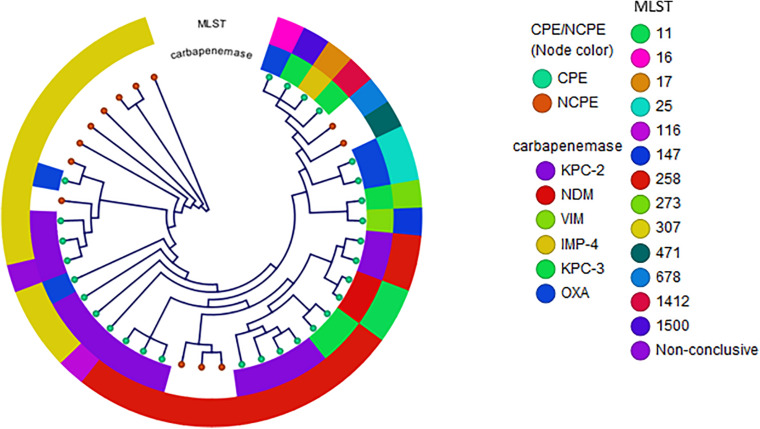
Phylogenetic reconstruction of *K. pneumoniae.* Distribution of isolates using SNP-based core genome alignments. Node color indicates CPE or NCPE. Layer 1 shows carbapenemase types. Layer 2 shows clonal groups by MLST.

Among *K. pneumoniae* isolates, 62% were CPE with KPC-2 as the most prevalent enzyme. The two most common clonal groups (CG) of *K. pneumoniae* were CG258 [14 (33%) of 42] and CG307 [14 (33%) of 42]. CG258 represented 43% of the carbapenemase-producing *K. pneumoniae* (Figure 1). Of the 14 CG258 *K pneumoniae*, 79% were carbapenemase-producing, harboring primarily *bla*_KPC–2_ (80%) or *bla*_KPC–3_ (20%). The other predominant clonal group was CG307, which demonstrated a lower rate of carbapenemase-producing isolates [5 (36%)] as compared to CG258. In CG307 strains, *bla*_KPC–2_ was the most common carbapenemase. One blood isolate from a patient who recently traveled to India harbored *bla*_OXA–232_. In addition, all CG307 carried *bla*_CTX–M_, a common group of extended-spectrum β-lactamases in this clonal group.

**FIGURE 2:**
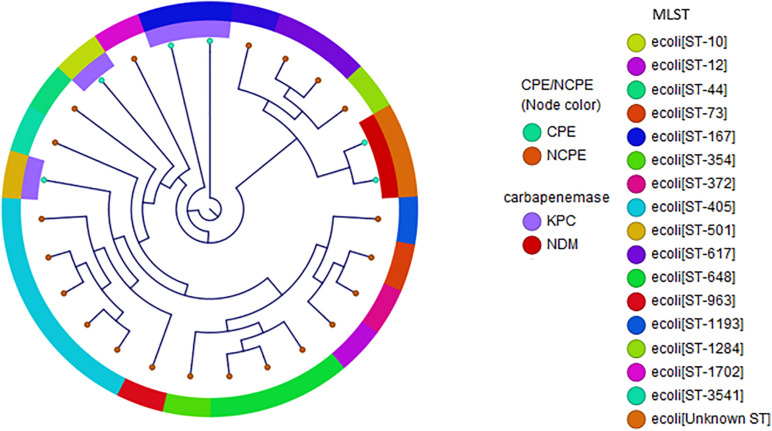
Phylogenetic reconstruction of *E. coli.* Distribution of isolates using SNP-based core genome alignments. Node color indicates CPE or NCPE. Layer 1 shows carbapenemase types. Layer 2 shows clonal groups by MLST.

In *E. coli*, diverse genetic lineages were observed (Figure 2). No carbapenemase genes were present in 75% of *E. coli*. Notably, while ST131 has been reported as a common clonal type in this species, it was not identified in this diverse set. The carbapenemases in *E. coli* were *bla*_KPC–2_ (*n* = 4), *bla*_KPC–3_ (*n* = 1), and *bla*_NDM_ (*n* = 2). Interestingly, two isolates containing *bla*_NDM–5_ were identified in two independent cases of patients from Mexico. Both cases were ambulatory with minimal exposures to the healthcare system in Mexico prior to hospitalization. One case had a bronchial alveolar lavage performed from which an acid-fast bacilli stain was positive and identified as *Mycobacterium tuberculosis*-complex by polymerase chain reaction (PCR) in addition to *E. coli.* The second case was from a urine culture with polymicrobial growth that detected *E. coli.* In both cases, detection of *bla*_NDM_ was made initially using the Cepheid Xpert^®^ Carba-R PCR test.

*Enterobacter cloacae* isolates were the third most frequent group of CRE. We observed diverse genetic heterogeneity consistent with *E. cloacae* complex. No carbapenemase genes were present in most isolates [9 (82%) of 11]. All but one isolate harbored *bla*_AmpC_ (*bla*_CMY_ and *bla*_ACT/MIR_) genes. Among these, six were found to have mutations in *ompF.* Among the two isolates with carbapenemase genes, one contained *bla*_KPC–3_ and one harbored *bla*_NDM–5_. The *bla*_NDM–5_ was identified from a case of a patient with recent travel from Guatemala and no other health care exposures or significant comorbidities. No molecular tests were used at time of clinical care; susceptibility patterns displayed resistance to carbapenems and ceftazidime-avibactam but susceptible to tigecycline.

### Epidemiology and Clinical Features

Phylogenetic reconstructions of *K. pneumoniae* and *E. coli* displaying types of NCPE and CPE demonstrated clonal heterogeneity. Three potential transmission clusters involving unique subjects were identified: one KPC-positive *K. pneumoniae* cluster (involving patients K702013 and K852013). Both isolates were *K. pneumoniae* ST307 [replicons IncFII(K)]. Another potential cluster of NCPE among three unique patients admitted on the same hospital floor was identified among *K. pneumoniae* ST307 (K2312011, K2542011, and K2342012).

Of the 53 individual patients with clinical data, 51% of patients were male and the median age was 55 years (interquartile range, 45–74 years; Table 4). Of the patients, 27 had acute care hospitalization and 26 had an emergency department visit in the prior 90 days. The majority of patients had antimicrobial use 90 days prior to culture. Approximately 47% (25/53) required admission to the intensive care unit. The all-cause 30-day mortality rate was 21% (11/53). At 30 days after culture date, 6% of patients were still hospitalized, 32% (17/53) were readmitted and were with lack of clinical response, 9% patients experienced relapse of their infection, 21% (11/53) remained on anti-CRE therapies, and 26% had developed worsening renal function. There were no differences in demographic or chronic conditions between patients with NCPE vs. CPE. Patients with NCPE (62%) were more likely to have had a prior ED visits as compared with patients with CPE (*p* = 0.011). The days of therapy (DOT) prior to culture was significantly higher among NCPE vs. CPE (795 vs. 242; *p* ≤ 0.001). There were no differences between these categories as compared to unconfirmed CREs. We found no significant difference between mortality (29% vs. 17%, CPE vs NCPE; *p* = 0.142).

**TABLE 4:** Clinical features of carbapenemase- and non-carbapenemase producing Enterobacterales.

	CPE (*n* = 17)	NCPE (*n* = 36)	*P*
Age (mean, SD)	49 (16)	51(11)	0.421
Male sex	10 (59)	17 (47)	0.212
Travel History	4 (23)	12 (33)	0.314
*Race/Ethnicity*			
White	12 (71)	28 (78)	0.570
Black	2 (12)	1 (3)	0.493
Hispanic	3 (18)	5 (14)	0.148
*Comorbidities*			
Charlson Comorbidity Score (mean, SD)	3.1 (0.6)	3.9 (0.8)	0.262
Myocardial infarct	0	0	–
Congestive heart failure	2 (12)	1 (3)	0.493
Peripheral vascular disease	5 (29)	17 (47)	0.352
Cerebrovascular disease	0	2 (6)	–
Dementia	0	0	–
Chronic pulmonary disease	2 (12)	2 (6)	0.809
Connective tissue disease	0	1 (3)	–
Ulcer disease	2 (12)	6 (17)	0.956
Mild liver disease	2 (12)	2 (6)	0.809
Diabetes (without complications)	3 (18)	6 (17)	0.761
Diabetes with end organ damage	1 (6)	6 (17)	0.517
Hemiplegia	0	4 (11)	–
Moderate or severe renal disease	6 (35)	11 (31)	0.976
Solid tumor (non metastatic)	1 (6)	5 (14)	0.693
Leukemia	1 (6)	0	–
Lymphoma, Multiple myeloma	0	0	–
Moderate or severe liver disease	2 (12)	1 (3)	0.493
Metastatic solid tumor	0	1 (3)	–
AIDS	0	0	–
HSCT – autologous	0	1 (3)	–
HSCT – allogenic	0	0	–
Hemodialysis	0	0	–
*Setting prior to admission*			
Home	12 (71)	25 (69)	0.932
Nursing Home/Long-term chronic care	1 (6)	7 (19)	0.198
Hospital Transfer	2 (12)	1 (3)	0.108
Long-term acute care	0	0	–
Transfer from different region/country	0	0	–
Hospice	0	0	–
*Prior hospitalization or ED visits past 90 days*			
Prior Hospitalization (>1)	6 (35)	21 (58)	0.146
Prior ED visits (>1)	4 (23)	22 (61)	0.011
*Prior Antibiotics in last 90 days*			
Fluoroquinolones	4 (23)	10 (28)	
3rd/4th Gen Cephalosporins	1 (6)	6 (17)	
1st Gen Cephalosporins	2 (12)	5 (14)	
Carbapenems	1 (6)	7 (19.4)	
Penicillins	0	9 (25)	
Macrolides	1 (6)	5 (14)	
Vancomycin	1 (6)	4 (11)	
Overall days of therapy	242	795	<0.001

*CPE, carbapenemase-producing Enterobacterales; NCPE, non-carbapenemase-producing Enterobacterales; AIDS, acquired immunodeficiency syndrome; HCST, hematopoietic stem cell transplantation; ED, emergency department.*

## Discussion

Carbapenem-resistant Enterobacterales presents a critical problem worldwide due to rapidly rising resistance rates and poor outcomes. The majority of efforts against CRE have focused on the epidemiology and outcomes in the context of CPE. However, in this cohort from South Texas, 48% of Enterobacterales that met the CDC definitions for CRE did not carry carbapenemase genes, and 17 were not carbapenem resistant upon repeat susceptibility testing. This has important implications for both clinical management as well as for epidemiologic reasons.

A recent multistate surveillance study estimated CRE rates of 2.93 per 100,000, with NCPE representing over 50% of the isolates and non-carbapenemase producing *K. pneumoniae* (NCPK) making up 58.6% of the total number of species (Duin et al., 2020). The current study found local rates of NCPK to be 27.6% of total NCPE. DOT were significantly higher in the NCPE group, indicating recent antimicrobial exposure as a contributing factor for the development of NCPE CRE. Furthermore, patients with NCPE were also associated with higher number of visits to the ED.

We report a high proportion of NCPE with varying mechanisms. In conjunction with porin loss or mutations, production of *bla*_ESBL_ and *bla*_AmpC_ have been shown to confer carbapenem-resistance, particularly in NCPK strains (Guh et al., 2015). This work validates this as *bla*_KPC_, *bla*_CTX–M_-variants, and *bla*_SHV_-variants were more abundant in the NCPK strains while *bla*_pAmpCs_ were more prevalent in other Enterobacterales (*E. coli* and *E. cloacae*). This work further confirms that both porin alterations and ESBL/AMPC production must be related responses to antimicrobial therapy in these strains. In NCPK, ertapenem and meropenem therapy failure have been shown to result from OmpK36 absence, with mutations playing a theoretical role (Hamzaoui et al., 2018). Moreover, *K. pneumoniae* outer membrane porin composition shifts during carbapenem therapy, specifically, the more restrictive OmpK36 porin seems to be favored over the wider OmpK35 porin in carbapenem-resistant strains (Findlay et al., 2012). The structure of OmpK36 is consistent with other Enterobacterales outer membrane proteins, consisting of 16 antiparallel β-barrel strands connected by short and long loops at the periplasmic and extracellular sides, respectively. However, wildtype OmpK36 extracellular loop 3 (L3) folds into the channel, partially obstructing the channel (Hasdemir et al., 2004). A solved crystal structure of OmpK36 from a meropenem-resistant strain has been shown to have a Gly115-Asp116 insertion within L3, stabilized by a salt bridge, further constricting meropenem’s uptake compared to wildtype OmpK36 (Dutzler et al., 1999). Additionally, a premature OmpK36 stop codon at K103stop has been previously characterized in this phenotype, eliminating the critical extracellular loops and resulting in a null porin (Wong et al., 2019). *K. pneumoniae* strains which produce Ompk37 in an Ompk35/36-null membrane, have been shown to significantly select against β-lactam entry while maintaining nutrient uptake (Ruiz et al., 2012). OmpK37 achieves this selectivity by way of an inward-folded extracellular L3 loaded with fully or partially charged residues and a strategically oriented Y118 residue, shielding the channel further (Supplementary Figure 1; Ruiz et al., 2012). Consistent among β-lactam-resistant strains, our strains maintained the Y118 residue and had mutations within L3 and the OmpK37 channel which made for a more hydrophobic environment. However, the single polar-aromatic to basic nucleotide mutation at the intracellular side of the channel, Y311H, that has been observed in a small number of meropenem-resistant strains previously but was not discovered in our strains (Wong et al., 2019).

Molecular analysis of this clinical collection uncovered several emerging trends in this region. We note that since 2013, CG307 was associated with as many CRE infections as those of the previous common clone, CG258. CG307 strains varied markedly in their gene content with carriage of *bla*_CTX–M_ and *bla*_KPC_ although were more likely to be NCPE compared to CG258. This is consistent with trends from the Houston region, supporting the regional introduction and spread of this novel high-risk clone (Duin et al., 2020). Moreover, we identified two cases of *K. michiganensis*, a relatively new identified pathogen that is commonly mis-identified by clinical laboratories as *Klebsiella oxytoca* (Pagès et al., 2008). An isolate in this study was found to harbor the plasmid-borne gene *mcr-9* associated with polymyxin resistance. This is the first study to describe *mcr-9* harboring *K. michiganensis* in TX, United States.

We identified five strains expressing *bla*_NDM_ that conferred broad resistance to nearly all β-lactams. Of these, two isolates harboring *bla*_NDM_ were discovered in patients with recent travel history from Mexico. Recent studies have described outbreaks of *bla*_NDM–1_ harboring nosocomial strains of *E. cloacae* and *K. pneumoniae* in Mexico City and other border cities (Founou et al., 2018). Global spread of *bla*_NDM_-positive plasmids is concerning as these strains are associated with extremely reduced antimicrobial treatment options.

While the high proportion of NCPE was surprising, one explanation of this trend might be driven by the updated CDC definition of CRE in 2015. Other reports demonstrated an increase in “CRE” with the application of these definitions. In addition, we describe unconfirmed cases that met the CDC-defined definition (Duin et al., 2020). This subgroup may reflect some errors and differences in susceptibility testing methods. Furthermore, the expansion of the definition to include non-susceptible ertapenem (MIC breakpoint of 0.5 μg/mL) and inconsistencies between state and CDC definitions may have further contributed to discordant results. These could have implications for clinical microbiology laboratories. For example, all isolates that were intermediate resistant to ertapenem only while susceptible to all other carbapenems did not have detectable carbapenemases by the Cepheid Xpert^®^ Carba-R PCR test. For this reason, our institutions do not routinely conduct these rapid molecular tests if non-susceptible to ertapenem only. The direct clinical impact of these various definitions, however, was not within the scope of this study. We found no significant difference between mortality between CPE vs NCPE. These findings are consistent with a recent prospective study found that clinical outcomes were not significantly different regardless of infection with CPE, NCPE, or unconfirmed CRE, with an overall mortality of 24% (Duin et al., 2020). Prior to the era of newer β-lactam/β-lactamase inhibitor combination, mortality rates from CRE reported to be up to 75% (Borer et al., 2009; van Duin et al., 2018; Wunderink et al., 2018; Alcántar-Curiel et al., 2019; Motsch et al., 2020).

There are several limitations to be noted in this study. First, information on clinical characteristics and outcomes could not be completely acquired for all unique patients because of the limitations of a retrospective study and migrating electronic health records. Second, the data herein is representative of two urban hospitals in South Texas; therefore, the generalizability is uncertain. However, it confers the importance of local/regional surveillance and epidemiology that may directly impact diagnostic and treatment practices. The variations in detection and antimicrobial susceptibility testing between institutions and with the research laboratory may have introduced varying results attributing to misattribution and overestimation of CRE. Carbapenems are very labile and are usually the first to fail in Vitek and disk diffusion. Therefore, procedures for automatic repeat testing with concurrent quality control and new reagents were performed to resolve discrepancies.

In summary, we demonstrate the wide heterogeneity of resistance mechanisms for CRE in this region with a predominance of NCPE. Identification and distinction of these mechanisms by rapid species identification and subsequent carbapenemase characterization would allow for optimal treatment and infection control efforts.

## Materials and Methods

### Bacterial Isolates

Ninety-nine CRE clinical isolates from unique patients at Methodist Hospital System and University Health System in San Antonio, TX, United States between 2011 and 2019 were included. The sources of the isolates included urine, blood, body fluid, pus, tracheal aspirate, sputum, wound swabs/tissue, central venous and arterial line tips. CRE was defined using the CDC definition (resistant to imipenem, meropenem, doripenem, or ertapenem OR documentation that the isolate possesses a carbapenemase) (Chea et al., 2015). Carbapenem non-susceptibility was defined based on CLSI breakpoints: meropenem MIC ≥ 2 μg/mL or ertapenem MIC ≥ 1 μg/mL (CLSI, 2018).

Carbapenem-resistant Enterobacterales with carbapenemase genes detected were termed CPE; those without carbapenemase genes were termed NCPE. We evaluated another category, termed unconfirmed-CRE, for CDC-defined CRE that although were identified as carbapenem-resistant by local laboratories, were found to be susceptible or intermediate to all tested carbapenems upon repeat testing.

### Antimicrobial Susceptibility Testing

Isolates tested non-susceptible by Vitek2^®^ (bioMérieux, Inc., NC, United States) or Etest^®^ (bioMérieux, Inc., NC, United States) per CLSI breakpoints at the time of isolation from clinical specimens were included (i.e., ertapenem MIC ≥ 1 μg/mL or meropenem, imipenem, doripenem MICs of ≥2 mg/L). *In vitro* susceptibility for 20 antimicrobials (Table 1) was repeated with Thermo Scientific Sensititre^TM^ GNX2F plate (Thermo Fisher Scientific, OH, United States) using broth microdilution method according to CLSI recommendations (M100-S28, 2018). Likewise, susceptibility was interpreted per CLSI except for colistin and tigecycline. CLSI epidemiological cutoff of ≥4 mg/L, which is also consistent with EUCAST resistant breakpoint of >2 mg/L, defined non-wild type for colistin and FDA breakpoints were used for tigecycline (i.e., Susceptible ≤2 mg/L, Intermediate 4 mg/L, Resistant ≥8 mg/L). *Pseudomonas aeruginosa* ATCC 27853 was used as a quality control organism.

### Clinical Data Collection

Medical records were reviewed to collect clinical and epidemiologic data, including sample date, location of culture collection, specimen source, patient demographics, travel history, health care exposures, underlying medical conditions, indwelling devices and antimicrobial therapy exposures 90 days prior to culture, infection types, clinical manifestations, and outcomes. This study was reviewed and approved by the UT Health San Antonio Institutional Review Board.

### DNA Sequencing and Analyses

Whole genome sequencing (WGS) was conducted on all 99 isolates using a NextSeq 500 sequencing instrument (Illumina Inc., San Diego, CA, United States) with 150-base paired-end reads (UT Health San Antonio, San Antonio, TX, United States). De-novo assembly and variant analyses were conducted using CLC Genomics Workbench 20.1 (Qiagen, Redwood City, CA, United States). The MLST database was used to identify the sequence types (STs) of the study isolates using WGS data. More detailed methods are provided in the Supplementary Material. Short-read data have been deposited in the NCBI BioProject (PRJNA688166).

### Statistical Analyses

The student *t*-test or the nonparametric Wilcoxon rank-sum test was used for continuous variables based on distribution. The χ^2^ or Fisher exact test was used to compare categorical variables. A two-sided *P*-value of less than 0.05 was considered statistically significant. All analyses were completed with SPSS 22 (IBMCorp).

## Data Availability Statement

The data presented in the study are deposited in the NCBI repository, accession number (PRJNA688166).

## Ethics Statement

The studies involving human participants were reviewed and approved by The University of Texas Health San Antonio. Written informed consent for participation was not required for this study in accordance with the national legislation and the institutional requirements.

## Author Contributions

GL: conceptualization. GL, CB, and WS: analyses and draft the manuscript. CB, SA, GG, RB, CC, and JS: data extraction and experimentation. GL, CB, WS, SD, and GG: data interpretation. SD, GG, and RB: provide edits. All authors contributed to the article and approved the submitted version.

## Conflict of Interest

The authors declare that the research was conducted in the absence of any commercial or financial relationships that could be construed as a potential conflict of interest.
