# Supplementary material for: Predominance of Non-carbapenemase Producing Carbapenem-Resistant Enterobacterales in South Texas
Source: Front Microbiol. 2021 Feb 10;11:623574. doi: 10.3389/fmicb.2020.623574 (PMC7902696; doi:10.3389/fmicb.2020.623574)
Supplement: Supplementary file 1 [file Data_Sheet_1.PDF]

Supplemental Materials

1.1 Bacterial isolates and microbiological methods

Carbapenem non-susceptibility was suspected when minimum inhibitory concentration (MIC) using automated testing systems (VITEK® 2 instrument) was >1 mg/L for imipenem or meropenem, or zone diameter on disc diffusion testing was ≤ 23 mm for imipenem or meropenem disk. Carbapenem MICs were confirmed by Etest (bioMerieux, France) with susceptibility defined according to Clinical & Laboratory Standards Institute (CLSI) breakpoints. A meropenem disc was used for subculture to prevent “plasmid dump” without selective pressure.

1.2 Methods and Analyses

The resistome was assembled by identifying predicted antimicrobial resistance determinants using the ResFinder, PointFinder, and ArgANNOT databases. For plasmid identification, de novo assembled contigs were searched against the PlasmidFinder database which were deemed highly similar to a reference plasmid when assembled contigs and sequences mapped with greater than 90% mutual coverage and 90% sequence identity to a reference plasmid.

Core genome alignments were generated (i.e. conserved orthologous regions present in all included genomes) for *K. pneumoniae* and *E. coli* isolates using input alignments from reference sequences (GenBank (*E. coli* NC_012966; *K. pneumoniae* CP000647). Sequences were then aligned to be concatenated into an artificial ‘genome assembly’, and used to create a Maximum Likelihood phylogenetic tree with 100 replicates for bootstrap value calculation. Metadata including MLSTs and carbapenemase-producing status were applied. We defined potential transmission clusters as 2 or more isolates that: (i) shared the same clonal group (ii) shared the same carbapenemase gene-positive plasmid (iii) epidemiologically linked by temporal and/or spatial associations and (ii) had pair-wise SNP distance less than 10.

1.3 Outcome Definitions

Outcome definitions were applied at 30 days from index culture date. Symptomatic response was defined as either resolution or improvement of symptoms at the time of discharge. Relapse was defined as a new culture episode from the same anatomical site with the same species of CRE within 30 days. Clinical response was defined as a symptomatic response without need for ongoing treatment with any antibiotics with in vitro activity against the index CDC-defined CRE and without relapse. Patients were deemed to have an adverse event if they had a *Clostridioides difficile* infection and/or post-culture renal failure.

Supplementary Figures

Figure S1. *K. pneumoniae* wildtype porin, OmpK37, with charged channel constriction residues highlighted. Left: View from extracellular face of OmpK37. Right: View from side of OmpK37 with the extracellular facing side oriented up. Basic residues are colored blue while polar-aromatic residues are colored green. Y188 is colored red. Y311 is colored magenta. PDB 6V78.

Figure S2. Phylogenetic analysis of clinical *K. michiganensis* and closely related *K.michiganensis* isolates (CGA_240325.1, CGA_276705.2).

Table S1. Distribution of carbapenem resistant *Enterobacterales* and source of infection

	Urine	Wound swab	Bone and soft tissues	Blood	Hepatobiliary specimen	Abdominal specimen	Respiratory specimens
Klebsiella pneumoniae	18	1	4	5	1	2	4
E. cloacae complex	3	0	3	0	1	0	2
Escherichia coli	10	0	2	2	3	3	4
Klebsiella aerogenes	1	0	0	0	1	2	2
Citrobacter freundii	1	0	1	0	0	1	0
Morganella morganii	1	0	1	0	0	0	0
Serratia marcescens	0	0	0	1	0	0	2
Klebsiella michiganensis	0	0	0	0	0	0	1
Hafnia alvei	1	0	0	0	0	0	0

Table S2. Resistome, plasmids, and molecular sequence type of *K. pneumoniae*

ID	CPE	Yr	MLST	Resistance	Plasmids	pmlsts
MK1	0	2018	ST-471	aac(6')Ib-cr,blaOXA-1,blaSHV-108-like,catB3-like,dfrA14-like,fosA-like,oqxA-like,oqxB-like,QnrB66-like,tet(A)	IncFII(pKPX1),IncFIB(K),IncFII(K)	IncF[[K7:A-:B-]]
H1092015	0	2015	Unknown	aadA1,aadA2,blaOXY-2-8-like,dfrA1,dfrA12,mph(A),sul1,tet(D)	IncFIB(pB171),IncHI1A(NDM-CIT),IncHI1B(pNDM-CIT)	IncHI1[Unknown ST],IncF[[F-:A-:B36*]]
H1112013	1	2013	Unknown	aac(6')-33,aac(6')Ib-cr-like,aadA2,aadB,blaCTX-M-15,blaKPC-2,blaOXA-1-like,blaOXA-9-like,blaSHV-1-like,blaTEM-1B-like,dfrA12,dfrA14-like,dfrA17-like,fosA-like,mph(A),oqxA-like,oqxB-like,strA-like,strB-like,sul1,sul2	IncFIB(K),IncFII(K)	IncF[[K2*:A-:B-]]
H11120132	1	2013	Unknown ST	aac(6')-33,aac(6')-Ib,aac(6')Ib-cr-like,aadA2,aadB,blaCTX-M-15,blaKPC-2,blaOXA-9-like,blaSHV-28,blaTEM-1B-like,dfrA12,dfrA14-like,dfrA17-like,fosA-like,mph(A),oqxA-like,oqxB-like,strA-like,strB-like,sul1,sul2	IncFIB(K),IncFII(K)	IncF[[K2*:A-:B27*]]
H1132015	0	2015	ST-307	aac(3)-IIa,aac(6')-Ib,aac(6')Ib-cr-like,aadB,blaCTX-M-15,blaSHV-28,blaTEM-1B-like,catB3-like,dfrA14-like,fosA-like,mph(A),mph(E),msr(E),oqxA-like,oqxB-like,strA-like,strB-like,sul1-like,sul2	FIA(pBK30683)	IncF[[F-:A13:B-]]
H1142013	1	2013	ST-307	blaCTX-M-15,blaKPC-2,blaOXA-9-like,blaSHV-28,blaTEM-1B-like,dfrA14-like,fosA-like,oqxA-like,oqxB-like,strA-like,strB-like,sul2	IncFIB(K),IncFII(K)	IncF[[K2*:A-:B-]]
H1192015	1	2015	ST-258	aac(6')-33,aac(6')-Ib,aac(6')Ib-cr-like,aadB,blaKPC-2,blaSHV-11,blaTEM-123-like,fosA-like,oqxA,oqxB,sul1,sul2		NA
H1620122	0	2012	ST-307	aac(3)-IIa,aac(6')-Ib,aac(6')Ib-cr-like,aadA2,aadB,blaCTX-M-15,blaFOX-5,blaSHV-28,catA2-like,catB3-like,dfrA12,dfrA14-like,fosA-like,mph(A),oqxA-like,oqxB-like,strA-like,strB-like,sul1,sul2,tet(D)	FIA(pBK30683)	IncF[[F-:A13:B-]]
H1792014	1	2014	ST-16	aac(6')Ib-cr-like,ARR-2,blaCTX-M-15,blaOXA-1,blaOXA-232,blaSHV-1,catB3-like,dfrA1,fosA-like,oqxA-like,oqxB-like,rmtf-like,sul1	IncFII(pKPX1),IncFIB(pKPHS1),IncFIB(K)	IncF[Unknown ST]
H1862013	1	2013	ST-258	aac(6')-33,aac(6')-Ib,aac(6')Ib-cr-like,aadB,blaKPC-2,blaSHV-11,blaTEM-123-like,fosA-like,oqxA,oqxB,sul1,sul2		NA
H1942017	0	2017	ST-307	aac(3)-IIa-like,aac(6')Ib-cr,blaCTX-M-15,blaOXA-1,blaSHV-28-like,blaTEM-1B,catB3-like,dfrA14-like,fosA-like,oqxA-like,oqxB-like,strA,strB,sul2	IncFIB(K),IncFII(K)	IncF[[K7:A-:B-]]
H1962011	0	2011	ST-307	aac(3)-IIa,aac(6')-Ib,aac(6')Ib-cr-like,blaCTX-M-15,blaSHV-28,catA2-like,dfrA14-like,fosA-like,oqxA-like,oqxB-like,strA-like,strB-like,sul2,tet(D)	FIA(pBK30683)	IncF[[F-:A13:B-]]

H1972015	0	2015	ST-678	blaSHV-41,fosA-like,oqxA-like,oqxB-like	IncFIB(K),IncFII(K),FII(pBK30683)	IncF[[K6*:A-:B-]]
H2002013	0	2013	ST-307	aac(3)-IIa,blaCTX-M-15,blaSHV-28,dfrA14-like,fosA-like,oqxA-like,oqxB-like,tet(D)	FIA(pBK30683)	IncF[[F-:A13:B-]]
H2172013	0	2013		aac(6')-IIc,aac(6')Ib-cr-like,aacA4,aadA1,aadA2,blaOXY-2-8-like,blaSHV-12,blaTEM-1B-like,catA2-like,dfrA1,dfrA18-like,ere(A)-like,strA,strB,sul1,sul2,tet(D)	IncHI2A,IncHI2	IncHI2[ST-1]
H2212017	0	2017	ST-25	aac(3)-IIa-like,aac(6')Ib-cr,blaCTX-M-15,blaOXA-1,blaSHV-11-like,blaTEM-1B,catB3-like,dfrA14-like,fosA-like,oqxA-like,oqxB-like,QnrB66-like,strA,strB,sul2,tet(A)	IncFIB(K),IncFII(K)	IncF[[K2:A-:B-]]
H2232017	1	2017	ST-258	aac(6')-Ib-like,aac(6')Ib-cr-like,blaKPC-2,blaLEN12-like,fosA-like,oqxA,oqxB	IncFIB(K),IncFIB(pQil),IncFII(Y)	IncF[[Y6:A-:B-]]
H2252017	1	2017	ST-258	aac(6')-Ib-like,aac(6')Ib-cr-like,blaKPC-2,blaLEN12-like,fosA-like,oqxA,oqxB	IncFIB(pQil),IncFIB(K),IncFII(Y)	IncF[[Y6:A-:B-]]
H2312011	0	2011	ST-307	aac(3)-IIa,aac(6')-Ib,aac(6')Ib-cr-like,blaCTX-M-15,blaSHV-28,catA2-like,dfrA14-like,fosA-like,oqxA-like,oqxB-like,strA-like,strB-like,sul2,tet(D)	FIA(pBK30683)	IncF[[F-:A13:B-]]
H2342012	0	2012	ST-307	aac(6')-Ib,aac(6')Ib-cr-like,blaCTX-M-15,blaSHV-28,blaTEM-1B-like,catA2-like,dfrA14-like,fosA-like,oqxA-like,oqxB-like,strA-like,strB-like,sul2,tet(D)	FIA(pBK30683)	IncF[[F-:A13:B-]]
H2372014	0	2014	ST-258	aac(6')-Ib,aac(6')Ib-cr-like,aadA2,blaCTX-M-15,blaSHV-5-like,blaTEM-1A-like,dfrA12,fosA-like,mph(A),oqxA,oqxB,sul1	IncFIB(pQil),IncFIB(K)	IncF[Unknown ST]
H2472012	1	2012	ST-11	aac(3)-IIa-like,aac(6')Ib-cr-like,aacA4-like,aadA2,blaCMY-6,blaCTX-M-15,blaNDM-1,blaOXA-1,blaSHV-11,blaTEM-1B-like,catA2-like,catB3-like,dfrA14-like,fosA-like,mph(A),oqxA,oqxB,QnrB58-like,rmtC,strA-like,strB-like,sul1,sul2,tet(A),tet(D)	IncFIB(K),IncFII(K),FIA(pBK30683)	IncF[[F-:A13:B-]]
H2542011	0	2011	ST-307	aac(3)-IIa,aac(6')Ib-cr-like,aacA4-like,blaCTX-M-15,blaSHV-28,catA2-like,dfrA14-like,fosA-like,oqxA-like,oqxB-like,strA-like,strB-like,sul2,tet(D)	FIA(pBK30683)	IncF[[F-:A13:B-]]
H2582017	0	2017	ST-25	aac(6')Ib-cr,blaCTX-M-15,blaOXA-1,blaSHV-11-like,catB3-like,dfrA14-like,fosA-like,oqxA-like,oqxB-like,QnrB66-like,tet(A)	IncFII(29),IncFIB(K)	IncF[[F29:A-:B-]]
H2742017	1	2017	ST-258	aac(6')-Ib-like,aac(6')Ib-cr-like,blaKPC-2,blaSHV-12,fosA-like,oqxA,oqxB	IncFIB(pQil),IncFIB(K),IncFII(Y)	IncF[[Y6:A-:B-]]
H2772017	1	2017	ST-258	aac(6')-Ib-like,aac(6')Ib-cr-like,blaKPC-2,blaSHV-12,fosA-like,oqxA,oqxB	IncFIB(pQil),IncFIB(K),IncFII(Y)	IncF[[Y6:A-:B-]]
H2872011	0	2011	ST-258	aac(6')-Ib,aac(6')Ib-cr-like,aadA2,blaCTX-M-15,blaSHV-12,blaTEM-1A-like,dfrA12,fosA-like,mph(A),oqxA,oqxB,sul1	IncFIB(K),IncFIB(pQil),IncFII(K)	IncF[[K7:A-:B-]]
H2962011	0	2011	ST-258	aac(6')-Ib,aac(6')Ib-cr-like,aadA2,blaCTX-M-15,blaSHV-12,blaTEM-1A-like,dfrA12,fosA-like,mph(A),oqxA,oqxB,sul1	IncFIB(pQil),IncFIB(K),IncFII(K)	IncF[[K7:A-:B-]]

H3092018	1	2018	ST-258	aac(6')-33,aac(6')-Ib,aac(6')Ib-cr-like,aadB,blaKPC-2,blaSHV-11,blaTEM-1D-like,fosA-like,oqxA,oqxB,sul1,sul2	IncFIB(K)	IncF[Unknown ST]
H3112018	1	2018	ST-1500	aac(6')-Ib,aac(6')Ib-cr-like,aadA1,aadB,blaKPC-3,blaSHV-30-like,dfrA1,dfrA14-like,fosA-like,oqxA-like,oqxB-like,QnrS1,sul1	IncFIB(K)(pCAV1099-114),IncN	IncN[ST-5],IncF[Unknown ST]
H312014	1	2014	ST-258	aac(6')-Ib-like,aac(6')Ib-cr-like,aadA1,aadA2,blaKPC-3,blaOXA-9,blaSHV-11,catA1-like,dfrA12,dfrA14-like,fosA-like,mph(A),oqxA,oqxB,strA,strB,sul1,sul2	IncFIB(K),FII(pBK30683)	IncF[[K3*:A-:B-]]
H3162018	1	2018	ST-258	aac(6')-Ib,aac(6')Ib-cr-like,blaKPC-2,blaOXA-9-like,blaSHV-11,blaTEM-1A-like,fosA-like,oqxA,oqxB	IncFIB(pQil),IncFII(K)	IncF[[K2:A-:B-]]
H3362019	1	2019	ST-1412	blaKPC-3,blaSHV-1,dfrA14-like,fosA-like,oqxA-like,oqxB-like,QnrS1	IncFIB(K),IncN	IncN[ST-6],IncF[Unknown ST]
H342014	1	2014	ST-258	aac(6')-Ib-like,aac(6')Ib-cr-like,aadA1,aadA2,blaKPC-3,blaOXA-9,blaSHV-11,catA1-like,dfrA12,dfrA14-like,fosA-like,mph(A),oqxA,oqxB,strA,strB,sul1,sul2	IncFIB(K),FII(pBK30683)	IncF[[K3*:A-:B-]]
H3732011	1	2011	ST-258	aac(6')-Ib,aac(6')Ib-cr-like,aadA2-like,blaKPC-2,blaOXA-9-like,blaSHV-11,blaTEM-1A-like,catA1-like,dfrA12,fosA-like,mph(A),oqxA,oqxB,sul1	IncFIB(K),IncFII(K)	IncF[[K1:A-:B-]]
H382012	1	2012	ST-307	aac(3)-IIa,blaCTX-M-15,blaKPC-2,blaOXA-9-like,blaSHV-28,dfrA14-like,fosA-like,oqxA-like,oqxB-like,strA-like,strB-like,sul2	IncFIB(pQil),IncFII(K),FIA(pBK30683)	IncF[[K2:A13:B-]]
H412014	0	2014	ST-307	aac(3)-IIa,aac(6')-Ib,aac(6')Ib-cr-like,blaCTX-M-15-like,blaOXA-9-like,blaSHV-28,dfrA14-like,fosA-like,oqxA-like,oqxB-like	IncFIB(pQil),IncFII(K),FIA(pBK30683)	IncF[[K2:A13:B-]]
H672012	1	2012	ST-147	aac(3)-IIa-like,aac(6')Ib-cr,aadA24-like,aph(3')-Ia,blaCTX-M-15,blaOXA-1,blaSHV-11-like,blaVIM-27,catB3-like,dfrA1,fosA-like,mph(A),oqxA-like,oqxB-like,strA,strB,sul1,tet(A)	IncHI1B(pNDM-MAR),IncFIB(pKPHS1),IncFII,IncFIB(pNDM-Mar)	IncHI1[Unknown ST],IncF[[F2:A-:B-]]
H682012	1	2012	ST-17	aac(3)-IId-like,aac(6')Ib-cr-like,aacA4-like,blaIMP-4,blaSHV-11,blaTEM-1B,catB3-like,fosA-like,mph(A),oqxA-like,oqxB-like,QnrB2,sul1-like	IncFII(K)	IncF[[K7:A-:B-]]
H692012	1	2012	ST-11	aac(3)-IIe-like,aac(6')Ib-cr-like,aacA4-like,aadA2,aadA5,blaCMY-6,blaCTX-M-15,blaDHA-1-like,blaNDM-1,blaOXA-1,blaSHV-11,blaTEM-1B,catA2-like,catB3-like,dfrA14-like,dfrA17,fosA-like,mph(A),oqxA,oqxB,QnrB58-like,rmtC,strA,strB,sul1,sul2,tet(A),tet(B)	IncFIB(K),IncFII(K),FIA(pBK30683)	IncF[[F-:A13:B-]]
H702013	1	2013	ST-307	aac(3)-IIa,blaCTX-M-15,blaKPC-2,blaOXA-9-like,blaSHV-28,dfrA14-like,fosA-like,oqxA-like,oqxB-like,strA-like,strB-like,sul2	IncFIB(pQil),IncFII(K),FIA(pBK30683)	IncF[[K2:A13:B-]]

H742014	1	2014	ST-307	aac(3)-IIa,aac(6')Ib-cr-like,aacA4-like,ARR-3-like,blaCTX-M-15-like,blaOXA-1,blaOXA-232,blaSHV-28,catB3-like,dfrA1,dfrA14-like,oqxA-like,oqxB-like,rmtf,sul1	IncFII(pKPX1),IncFIB(pKPHS1),IncFIB(K),FIA(pBK30683)	IncF[[K5*:A13:B-]]
H852013	1	2013	ST-307	aac(3)-IIa,aac(6')Ib-cr-like,aacA4-like,aadA1-like,aph(3')-Ic-like,armA,blaADC-25-like,blaCTX-M-15,blaKPC-2,blaOXA-66,blaOXA-72,blaOXA-9-like,blaSHV-28,catB8,dfrA14-like,fosA-like,mph(E),msr(E),oqxA-like,oqxB-like,strA-like,strB,sul1,sul2,tet(B)-like	IncFIB(pQil),IncFII(K),FIA(pBK30683)	IncF[[K2:A13:B-]]

Table S3. Resistome, plasmids, and molecular sequence type of *E. coli*

ID	Yr	MLST	Resistance	Plasmids	pmlsts
MP1	2018	ST-354	blaCTX-M-27,blaTEM-1B,dfrA17,strA,strB,sul2,tet(B)	IncFIA,IncFIB(pB171)	IncF[[F-:A1:B32]]
H1022013	2013	ST-648	aac(3)-IId-like,aadA5,blaCTX-M-14,blaTEM-1B,dfrA17,erm(B)-like,mph(A),strA,strB-like,sul1,sul2,tet(A)	IncFII(pRSB107),IncFIB(AP001918)	IncF[[F1:A-:B20]]
H1042012	2012	ST-3541	aadA5,blaCMY-59,blaCTX-M-15,blaTEM-1B-like,dfrA17,mph(A),sul1,tet(B)	IncFII,IncFIB(H89-PhagePlasmid),IncFIB(pB171)	IncF[[F36:A-:B32]]
H1312014	2014	ST-405	aac(3)-IIa-like,aadA5,blaCTX-M-14,blaCTX-M-15,blaTEM-1B,dfrA17,mph(A),strA,strB-like,sul1,sul2,tet(A)	IncFII,IncFIB(AP001918),IncFII(Y)	IncF[[F36:A-:B6]]
H1612014	2014	ST-405	aac(3)-IIa-like,aadA5,blaCTX-M-15,dfrA17,floR-like,mph(A),strA,strB,sul1,sul2-like,tet(A)-like,tet(B)	IncFII,IncFIB(H89-PhagePlasmid),IncFIB(AP001918)	IncF[[F31:A-:B10]]
H1712014	2014	ST-405	aac(3)-IIa-like,aadA5,blaCTX-M-14,blaCTX-M-15,blaTEM-1B,dfrA17,mph(A),strA,strB-like,sul1,sul2,tet(A)	IncFII,IncFIB(AP001918),IncFII(K)	IncF[[F31:A-:B6]]
H1752012	2012	ST-1284	aac(6')Ib-cr,aadA5,blaCTX-M-15,blaOXA-1,catB3-like,dfrA17,mph(A),sul1,tet(B)	IncFII,IncFIB(AP001918),IncFIA	IncF[[F36:A4:B1]]
H1792015	2015	ST-372	aadA1,blaTEM-1A,dfrA1,strA,strB,sul1	IncFIB(AP001918)	IncF[[F-:A-:B10]]
H1882013	2013	ST-617	aac(3)-VIa-like,aadA5,blaCMY-2,blaTEM-1C,dfrA17,erm(B)-like,floR-like,mph(A),strA,strB,sul1,sul2,tet(A)-like,tet(M)-like	IncFII,IncFIB(AP001918),IncFIA	IncF[[F36:A4:B1]]
H1992013	2013	ST-617	aac(3)-VIa-like,aadA5,aph(3')-Ia-like,blaCMY-2-like,blaTEM-1C,dfrA17,erm(B)-like,floR-like,mph(A),strA,strB,sul1,sul2,tet(A)-like,tet(M)-like	IncFII,IncFIB(AP001918),IncFIA	IncF[[F31:A4:B1]]
H2222015	2015	ST-12	aadA1,blaSHV-1-like,sul1	IncFII,IncFIB(AP001918)	IncF[[F51:A-:B10]]

H2632011	2011	ST-44	aac(6')Ib-cr,aadA5,blaCTX-M-15,blaOXA-1,catB3-like,dfrA17,mph(A),strA,strB,sul1,sul2	IncFII,IncFIA	IncF[[F36:A4:B-]]
H2632013	2013	ST-648	aac(3)-IId-like,aadA5,blaCTX-M-14,blaTEM-1B,dfrA17,erm(B)-like,mph(A),strA,strB-like,sul1,sul2,tet(A)	IncFII(pRSB107),IncFIB(AP001918),IncFII(pCoo)	IncF[[F1:A-:B20]]
H2672011	2011	ST-167	aac(3)-IId-like,aac(6')Ib-cr,aadA5,blaCTX-M-15,blaKPC-2,blaOXA-1,blaTEM-195-like,catB3-like, dfrA17, mph(A), sul1,tet(A)	IncFII,IncFIB(AP001918),IncFIA	IncF[[F36:A4:B1]]
H302013	2013	ST-167	aac(3)-IId-like,aac(6')Ib-cr,aadA5,blaCTX-M-15,blaKPC-2,blaOXA-1,blaTEM-1A-like,catB3-like,dfrA17,mph(A),sul1,tet(A)	IncFII,IncFIB(AP001918),IncFIA	IncF[[F36:A4:B1]
H3022017	2017	ST-501	aadA5,blaCTX-M-15,blaKPC-2,blaOXA-9-like,blaTEM-1A-like,dfrA17,mph(A)	IncFIB(AP001918),IncFII,IncFIB(pQil),IncFII(K)	IncF[[F24:A-:B1]]
H3072018	2018	ST-648	aac(3)-IIa-like,aac(6')Ib-cr,aadA5,blaCMY-2,blaCTX-M-15,blaOXA-1,catB3-like,dfrA17,mph(A),sul1,tet(A),tet(B)	IncFII(pRSB107),IncFIA,IncFIB(AP001918)	IncF[[F1:A1:B1]]
H3262013	2013	ST-405	blaCTX-M-15,strA,strB,sul2,tet(B)	IncFII(pRSB107),IncFIA	IncF[[F1:A1:B-]]
H3412019	2019	Unknown ST	aac(3)-IIa-like,aac(6')Ib-cr,aadA2,blaCTX-M-15,blaNDM-5,blaOXA-1,blaSHV-27-like,blaTEM-1B,catB3-like,dfrA12,dfrA14-like,fosA-like,mph(A)-like,oqxA-like,oqxB-like,QnrB66-like,strA,strB,sul1,sul2,tet(A)	IncFIB(K),IncFII,IncFIB(AP001918),IncFIA,IncFII(K)	IncF[[F36:A1:B1]]
H3422019	2019	Unknown ST	aac(3)-IIa-like,aac(6')Ib-cr,aadA2,blaCTX-M-15,blaNDM-5,blaOXA-1,blaSHV-27-like,blaTEM-1B,catB3-like,dfrA12,dfrA14-like,fosA-like,mph(A)-like,oqxA-like,oqxB-like,QnrB66-like,strA,strB,sul1,sul2,tet(A)	IncFIB(AP001918),IncFII,IncFIB(K),IncFII(K),IncFIA	IncF[[F36:A1:B1]]
H3722011	2011	ST-73			
H52014	2014	ST-10	aac(6')-Ib-like,aac(6')Ib-cr-like,aadA1,blaKPC-3,blaTEM-1B,dfrA14-like,QnrS1,strA,strB,sul2,tet(A)	IncN	IncN[ST-6]
H662014	2014	ST-405	aac(3)-IIa-like,aac(6')Ib-cr,aadA5,blaCTX-M-15,blaOXA-1,catB3-like,dfrA17,mph(A),sul1,tet(B)	IncFIB(AP001918)	IncF[[F-:A-:B10]]
H902013	2013	Unknown ST	aac(3)-IIa-like,aac(6')Ib-cr,aadA5,aph(3')-Ia,blaCMY-2,blaCTX-M-15,blaOXA-1-like,blaSHV-11,blaTEM-1B,catA1-like,catB3-like,dfrA14-like,dfrA17,erm(B)-like,fosA-like,mph(A),oqxA-like,oqxB-like,QnrB66-like,strA,strB-like,sul1,sul2,tet(B)	IncFII(K),FIA(pBK30683),IncFII,IncFIB(AP001918),IncFIA,IncFIB(K)	IncF[[F36:A1:B1]]

H972014	2014	ST-963	blaCMY-2	IncFII(pCoo)	IncF[[F96:A-:B-]]
H982014	2014	ST-1702	aac(6')Ib-cr,aadA5,blaCMY-42,blaCTX-M-15,blaOXA-1,catB3-like,dfrA17,mph(A),sul1,tet(A)	IncFII,IncFIA	IncF[[F36:A4:B-]]
H992014	2014	ST-1193	blaCTX-M-14	IncFIA,IncFIB(AP001918)	IncF[[F-:A1:B10]]

Table S4. Sequencing Outputs

ID	Org.	Gen. size	contigs	n50
MP1	E. coli	5011822	197	104768
H1022013	E. coli	5265795	301	97911
H1042012	E. coli	4983642	273	101439
H1312014	E. coli	5260158	328	56728
H1612014	E. coli	5281882	370	58261
H1712014	E. coli	5191813	387	51580
H1752012	E. coli	4921952	283	48856
H1792015	E. coli	4894733	247	104451
H1882013	E. coli	5102251	373	48465
H1992013	E. coli	5098968	386	51010
H2222015	E. coli	5125886	268	101937
H2632011	E. coli	4791908	292	60870
H2632013	E. coli	5391851	435	93921
H2672011	E. coli	5121873	253	69401
H302013	E. coli	5037530	275	57037
H3022017	E. coli	5250961	416	62680
H3072018	E. coli	5309552	257	77788
H3262013	E. coli	5168747	314	54127
H3412019	E. coli	10072149	810	37459
H3422019	E. coli	10070448	782	39282
H3722011	E. coli	5095221	303	99241
H52014	E. coli	4791081	214	85919

H662014	E. coli	5267140	457	64204
H902013	E. coli	10067243	1336	23328
H972014	E. coli	5142578	295	79243
H982014	E. coli	5030612	305	54698
H992014	E. coli	5080522	273	103146
MK1	K. pneumoniae	5618041	194	97790
H1092015	K. pneumoniae	6391692	230	87263
H1112013	K. pneumoniae	6511723	1539	14990
H11120132	K. pneumoniae	7383402	4221	12195
H1132015	K. pneumoniae	5480104	213	91052
H1142013	K. pneumoniae	5457482	208	86420
H1192015	K. pneumoniae	5682747	205	82271
H1620122	K. pneumoniae	5559348	219	86422
H1792014	K. pneumoniae	5731438	244	110928
H1862013	K. pneumoniae	5691318	227	95522
H1942017	K. pneumoniae	5493197	167	93424
H1962011	K. pneumoniae	5406599	203	95133
H1972015	K. pneumoniae	5473275	216	82319
H2002013	K. pneumoniae	5376413	183	95133
H2172013	K. pneumoniae	6307898	228	76756
H2212017	K. pneumoniae	5491932	276	87118
H2232017	K. pneumoniae	5535252	240	78669
H2252017	K. pneumoniae	5536586	238	84464
H2312011	K. pneumoniae	5406638	202	96619

H2342012	K. pneumoniae	5375663	201	81304
H2372014	K. pneumoniae	5423990	277	104214
H2472012	K. pneumoniae	6145583	838	22277
H2542011	K. pneumoniae	5406984	198	96648
H2582017	K. pneumoniae	5568366	149	100379
H2742017	K. pneumoniae	5538065	252	82273
H2772017	K. pneumoniae	5536857	242	92885
H2872011	K. pneumoniae	5380229	218	98041
H2962011	K. pneumoniae	5379422	216	98041
H3092018	K. pneumoniae	5705765	202	99686
H3112018	K. pneumoniae	5712099	750	107699
H312014	K. pneumoniae	5674308	257	88897
H3162018	K. pneumoniae	5457471	178	104506
H3362019	K. pneumoniae	5365762	126	115746
H342014	K. pneumoniae	5673905	264	89341
H3732011	K. pneumoniae	5605017	225	100381
H382012	K. pneumoniae	5490443	223	86277
H412014	K. pneumoniae	5474940	247	78267
H672012	K. pneumoniae	5656766	167	94000
H682012	K. pneumoniae	5437179	115	127858
H692012	K. pneumoniae	9450894	658	39687
H702013	K. pneumoniae	5501469	224	85719
H742014	K. pneumoniae	6237134	1013	20490
H852013	K. pneumoniae	9442217	444	85557
