# Supplementary figures and images for: Predominance of Non-carbapenemase Producing Carbapenem-Resistant Enterobacterales in South Texas
Source: Front Microbiol. 2021 Feb 10;11:623574. doi: 10.3389/fmicb.2020.623574 (PMC7902696; doi:10.3389/fmicb.2020.623574)

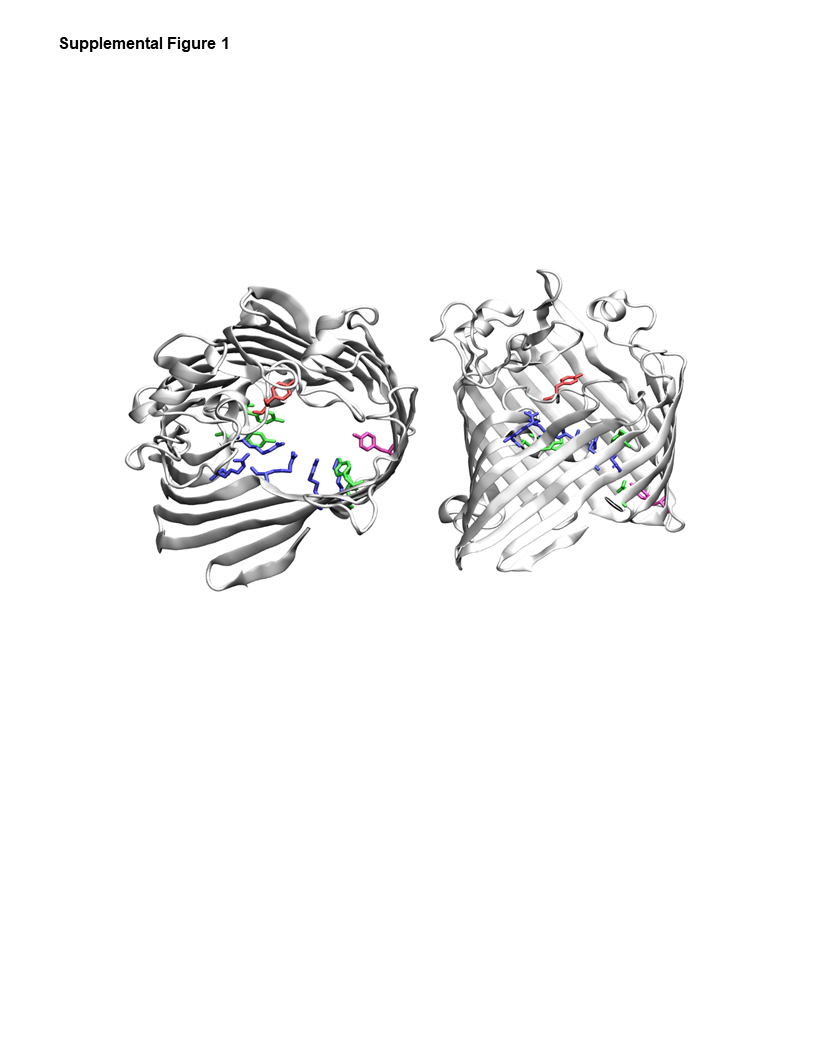

Supplement: Supplementary file 2 [file Image_1.TIF]

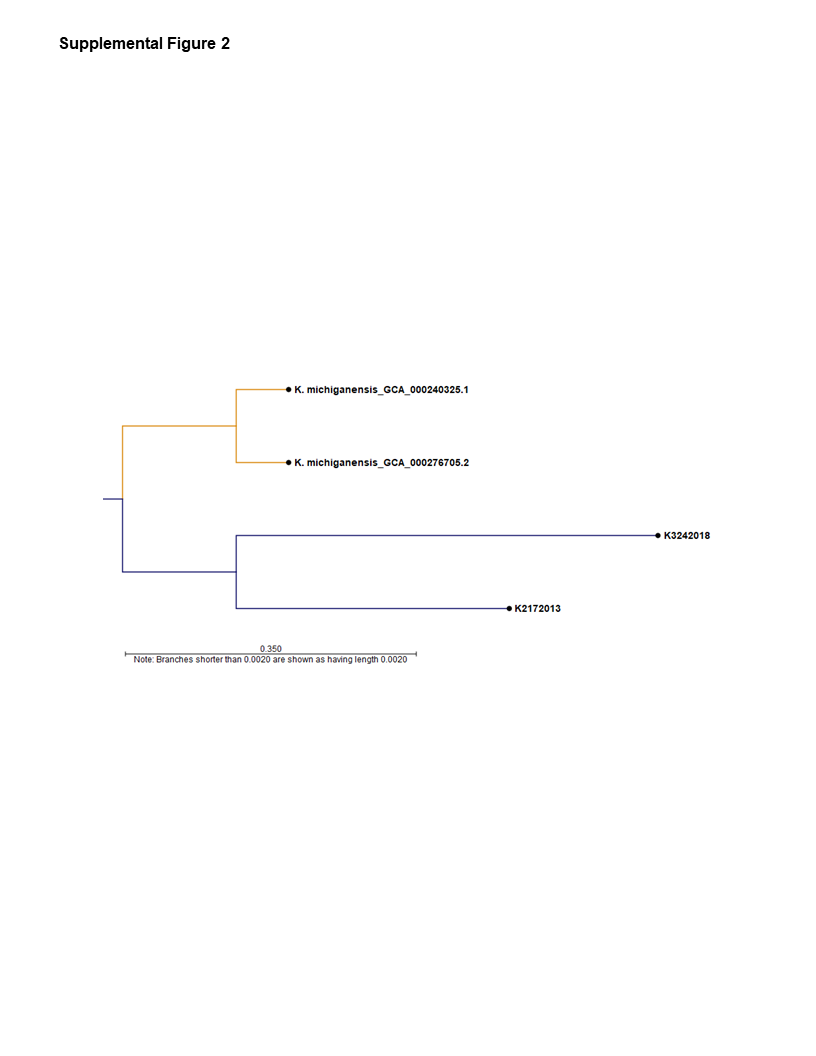

Supplement: Supplementary file 3 [file Image_2.TIF]
